# Timbre Encoding in the Inferior Colliculus

**DOI:** 10.1523/JNEUROSCI.1104-25.2026

**Published:** 2026-04-21

**Authors:** Johanna B. Fritzinger, Laurel H. Carney

**Affiliations:** ^1^Departments of Neuroscience, University of Rochester, Rochester, New York 14642; ^2^Biomedical Engineering, University of Rochester, Rochester, New York 14642

**Keywords:** auditory, auditory midbrain, computational models, inferior colliculus, timbre

## Abstract

Timbre, or the quality of a sound, is a critical component in speech and music. One percept of timbre, brightness, is correlated with the spectral centroid and the spectral envelope of harmonic sounds. Little is known about how this aspect of timbre is encoded in the subcortical auditory system. We used physiological and computational modeling methods to investigate the representation of spectral peaks in a harmonic complex tone with a broad, triangular-shaped spectrum. Extracellular single-neuron recordings were made in the central nucleus of the inferior colliculus (IC) in awake, female Dutch-belted rabbits. A population response to the timbre stimulus was inferred by shifting the stimulus spectrum above and below the characteristic frequency of each neuron. Spectral peaks in the stimulus were encoded in peaks in the average-rate profiles of most neurons, and this representation was robust over a range of suprathreshold levels. Neural discrimination thresholds were also sufficient to describe human behavioral thresholds. Temporal responses were complex and often exhibited phase locking to the fundamental frequency and integer multiples of the fundamental frequency. Computational models that included neural fluctuation sensitivity and amplitude modulation-sensitive broadband inhibition captured the major trends in physiological results. These findings demonstrate that multiple mechanisms may influence robust spectral peak encoding in IC neurons.

## Significance Statement

Spectral features, such as the spectral centroid and spectral peaks, are critical features of sounds with timbre, including speech and music. This study addresses a critical gap in knowledge about timbre representation in the auditory system by investigating neural encoding of spectral peaks in the inferior colliculus. Results demonstrate that spectral peaks are robustly represented in average-rate responses across sound levels and that neural discrimination thresholds are on a par with human thresholds. We found that a computational model with broad inhibition that is sensitive to neural fluctuations in peripheral responses may underly spectral peak encoding. These findings extend previous vowel encoding studies to provide broader insights into timbre processing, with potential applications for improving hearing-aid and cochlear-implant technologies.

## Introduction

**Figure 1.**
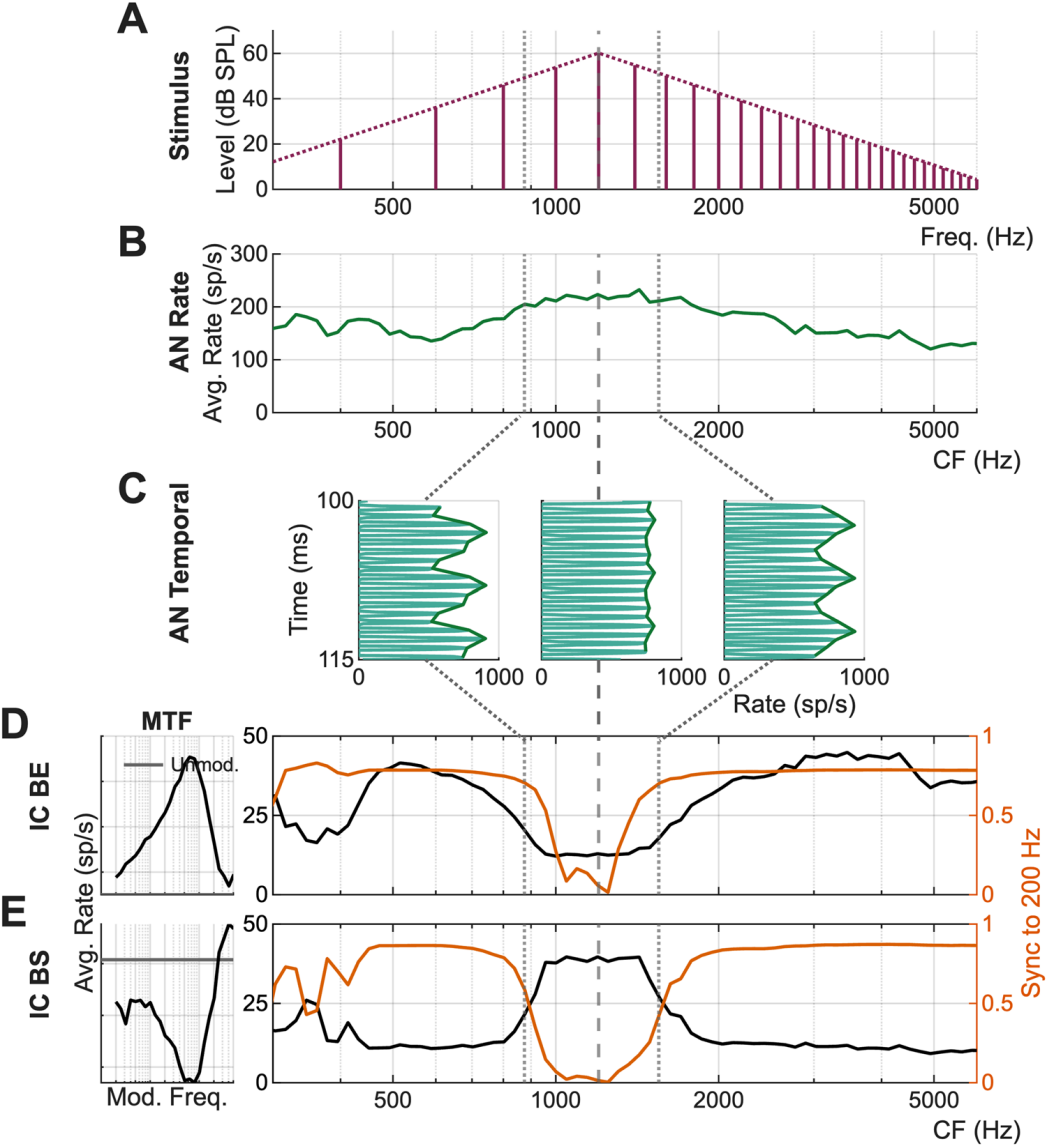
Illustration of rate and temporal coding of a spectral peak based on model AN and IC neurons. ***A***, Synthetic-timbre stimulus with spectral peak at 1,200 Hz (dashed line) and *F*_0_ of 200 Hz. ***B***, AN average-rate profile for a population of fibers with characteristic frequencies (CFs) spanning the stimulus spectrum. ***C***, Temporal responses for AN fibers tuned to 875, 1,200, and 1,550 Hz (dotted and dashed lines). ***D***, Population response for SFIE model IC BE neurons (MTF, left; Nelson and Carney, 2004). ***E***, Population response for model IC BS neurons (MTF, left). ***D, E***, Rate response (black) and synchronization coefficient to *F*_0_ = 200 Hz (orange).

Timbre is the sound quality that allows listeners to distinguish between complex sounds that are identical in pitch, duration, and loudness (ANSI, 1994). The percept of timbre contributes to speech intelligibility, music enjoyment, and sound-source recognition (Review: McAdams 2019). Timbre can be decomposed into spectral features (e.g., spectral centroid, slope, and variation) and temporal features (e.g., attack time; Grey and Gordon, 1978; Iverson and Krumhansl, 1993; McAdams et al., 1995). Timbre perception has been studied psychophysically; however, subcortical encoding of timbre remains poorly understood. This study used complex tones with triangularly shaped spectra (Fig. 1*A*) that elicit a timbral “brightness” percept (Allen and Oxenham, 2014) to test the hypothesis that spectral peaks, related to the spectral centroid, are robustly encoded in inferior colliculus (IC) rate and temporal response profiles (Fig. 1).

Although psychophysical timbre dimensions have not been studied in the IC, vowel encoding research gives insight into spectral peak encoding (Perez et al., 2013; Carney et al., 2015). Vowels are harmonic complexes with steeply sloping, triangular spectral peaks, steeper but otherwise similar to the stimuli used here (Lyzenga and Horst, 1995; Tan and Carney, 2005; Henry et al., 2017). Model and physiological responses to vowels in quiet support a transformation from temporal to rate coding due to amplitude modulation (AM) sensitivity of IC rates (Carney et al., 2015; Carney, 2018; Carney and McDonough, 2019). In background noise, temporal coding in the IC, which is excellent for periodic sounds, such as vowels or low-frequency AM stimuli (Rees and Langner, 2005; Kim et al., 2020), explains behavioral discrimination thresholds (Henry et al., 2017).

AM sensitivity in the IC was hypothesized to encode spectral peaks by providing sensitivity to neural fluctuation (NF) profiles in auditory nerve (AN) responses. NFs are low-frequency changes in the AN rate that vary in depth due to nonlinear inner-hair-cell (IHC) transduction (Carney, 2018, 2024). The NF depth for fibers tuned near spectral peaks would be reduced due to IHC saturation (Fig. 1*C*, middle). AN fibers tuned away from the spectral peak, where IHCs would not be saturated, would have deeper NFs (Fig. 1*C*, left, right). In the IC, band-enhanced (BE) neurons (Fig. 1*D*) or band-suppressed (BS) neurons (Fig. 1*E*) could encode spectral peaks in their average-rate or temporal responses due to the variation in NF depth across the spectral peak; BE cells would be suppressed at the spectral peak, whereas BS cells would be excited (Mao and Carney, 2015; Carney, 2018, 2024; Carney and McDonough, 2019; Maxwell et al., 2020; Fan et al., 2021).

AM-tuned broad inhibition, from frequencies spanning CF, could sharpen rate profiles at spectral peaks, as found in responses to tones in wideband noise (Fritzinger and Carney, 2025). Inhibition strongly impacts IC representations of complex sounds; blocking inhibition broadens tuning curves, removes frequency-sweep direction selectivity, and alters binaural responses (Andoni et al., 2007; Pollak, 2011; Williams and Fuzessery, 2011). Importantly, IC rate responses to tones in wideband noise can be described by a model with broadband modulation-sensitive inhibition (BMSI), suggesting that NF sensitivity is a driver of these responses. If AM-tuned broad inhibition is a stronger encoding mechanism than on-CF NFs alone, IC rate responses to spectral peaks may not depend simply on the modulation transfer function (MTF) type (Fritzinger and Carney, 2025).

This study investigated IC encoding of spectral peaks in synthetic-timbre stimuli by recording single-neuron responses in awake Dutch-belted rabbits and shifting the stimulus spectrum with respect to CF to infer population responses. Temporal patterns were more complex than predicted, exhibiting phase locking to the fundamental frequency (*F*_0_) and to integer multiples of *F*_0_. IC rate profiles robustly encoded spectral peaks at suprathreshold levels. IC rate-based neural timbre-discrimination thresholds, which depend on the temporal properties of IC inputs, were sufficient to explain human timbre-discrimination thresholds. Lastly, receptive field-based models of the rate profiles versus peak frequency revealed an impact of inhibition on rate profiles. A BMSI computational model accurately predicted most IC rate responses.

## Materials and Methods

### Surgical procedures

All procedures were approved by the University of Rochester Committee on Animal Resources in compliance with National Institutes of Health Guidelines. Four female Dutch-belted rabbits (*Oryctolagus cuniculus*) ranging from 6 months to 5 years of age were studied. All animals had normal hearing, monitored using distortion product otoacoustic emissions (Whitehead et al., 1992).

Animals were anesthetized intramuscularly with 66 mg/kg ketamine and 2 mg/kg xylazine or 35 mg/kg ketamine and 0.10–0.15 mg/kg dexmedetomidine for headbar placement, initial craniotomy and microdrive placement, and microdrive replacements. The headbar placement surgery consisted of affixing a custom 3D-printed headbar (Protolabs) to the skull of the animal using screws and dental acrylic. The headbar was designed with a chamber to hold a microdrive. After recovery, a craniotomy was performed, and a custom microdrive containing four tetrodes, modeled after the Neuralynx Five-drive microdrive (Neuralynx), was inserted into the headbar. The microdrive consisted of a 3D-printed body with stainless-steel guide tubes that extended into the craniotomy. Microdrive replacement surgeries were performed, on average, every 6 months.

Experiments were discontinued if emissions dropped by 10 dB or if neural thresholds at CF exceeded 50 dB SPL. Animals were euthanized, and the IC was sectioned and stained to verify tetrode location in the central nucleus.

### Stimulus presentation and neural-recording procedure

Each recording session was 2 h in duration inside a sound-attenuated booth (Acoustic Systems). Animals were head-fixed using the headbar and placed in a custom chair and wrapped in a towel to limit movement. Custom earmolds (Hal-Hen Company or Dreve Otoform Ak) were inserted for closed-field stimulus presentation.

Stimuli were generated using MATLAB, sent to an audio interface (16A, Mark of the Unicorn), and converted from digital to analog (DAC3 HGC, Benchmark Media Systems). Stimuli were then presented using earphones (Beyerdynamic DT-48, beyerdynamic, or Etymotic ER2, Etymotic Research). At the beginning of each session, a calibration curve was calculated by presenting 50–20 kHz tones and recording the speaker output using a probe-tube microphone (Etymotic ER10B + or ER7C, Etymotic Research). The stimuli were filtered by the calibration curve to compensate for the frequency response of the acoustic system.

Neural recordings were made with tetrodes consisting of four twisted 18 μm epoxy-coated platinum-iridium wires (California Fine Wire) plated with a platinum-black solution (Neuralynx) to lower impedances to ∼0.1–1.5 MΩ. Neural signals from the tetrodes were recorded using a 16-channel headstage, amplifier, and software from the Intan RHD recording system (Intan Technologies). Tetrodes were advanced and retracted at the beginning or end of a session to sample different areas of the IC. The location of the recordings was determined to be the central nucleus of the IC if tuning increased with tetrode depth.

After recording, waveforms were filtered and sorted into single-unit neurons as described below. First, waveforms were filtered using a fourth-order Butterworth bandpass filter with passband from 300 to 3,000 Hz. Second, a threshold of four times the standard deviation (SD) of the waveforms was set to determine spike times (Quiroga et al., 2004). Lastly, the waveforms were sorted into neurons using the custom spike-sorting software (Schwarz et al., 2012). A waveform had to meet two criteria to be considered a single-unit neuron: <2% of spikes occurred with intervals shorter than 1 ms, and the cluster entanglement metric was <0.1 (Schwarz et al., 2012). The cluster entanglement metric assesses separability of neurons using the L-ratio, a Mahalanobis distance-based metric that quantifies cluster contamination (Schmitzer-Torbert et al., 2005).

### Stimuli and analysis

At the beginning of each recording session, a set of standard stimuli were presented to characterize each neuron. This characterization included estimating the best frequency of a neuron over many sound levels by presenting pure-tone stimuli with varying frequencies and sound levels. The pure tones were presented from 250 to 16,000 Hz in five steps per octave at 10, 30, 50, and 70 dB SPL. The stimuli were 200 ms in duration with 10 ms raised-cosine on/off ramps and were presented diotically for three repetitions in a random order, with 400 ms interstimulus intervals. The CF was estimated as the frequency that elicited the largest average-rate response at the lowest sound level.

MTFs, to assess AM sensitivity, were measured by presenting broadband Gaussian noise (100−10 kHz) sinusoidally modulated from 2 to 600 Hz, with three steps per octave. Stimuli were presented at the 33 dB SPL spectrum level and were 1 s in duration with 50 ms raised-cosine on/off ramps, with 500 ms interstimulus intervals. Stimuli were presented five times, diotically, in a random sequence. MTFs were classified into four categories based on the average-rate responses to modulated noises compared with an unmodulated noise baseline (Fritzinger and Carney, 2025). Neurons were classified as BE if two modulation-frequency rates were significantly higher than the unmodulated rate, without a rate at an intermediate modulation frequency that was significantly below the unmodulated rate. Categorization for BS neurons required two rates that were significantly below the unmodulated rate, without an intermediate response that exceeded the unmodulated response. Hybrid neurons satisfied both BE and BS criteria, at different modulation frequencies, and flat neurons did not satisfy either criteria.

**Figure 2.**
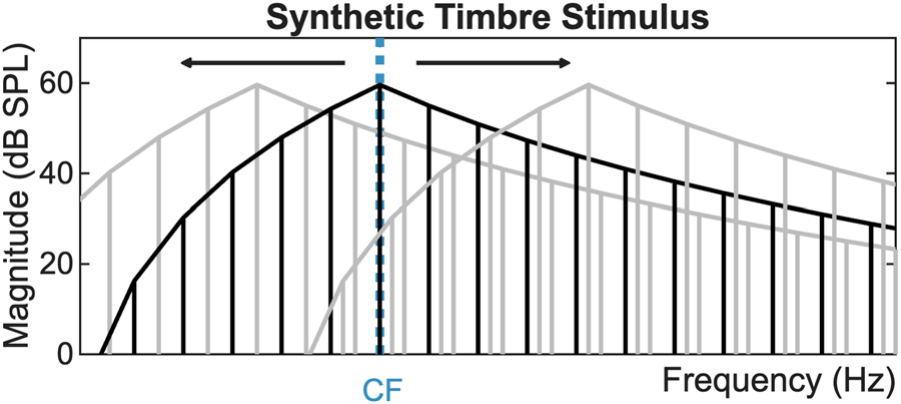
Schematic of shifted synthetic-timbre stimulus with triangular spectral envelope. The peak frequency of the reference stimulus (black) was the multiple of 200 Hz closest to the CF of the neuron.

The synthetic-timbre reference stimulus was based on Allen and Oxenham (2014) and consisted of a harmonic complex tone with 200 Hz *F*_0_ and a triangular-shaped spectrum with a roll-off of 24 dB/octave. The peak harmonic in the reference stimulus was the multiple of *F*_0_ nearest to the CF of the neuron. All harmonic components were added in the sine phase. The upper frequency limit of the stimulus was 10 kHz. The stimulus was 300 ms duration with 20 ms raised-cosine on/off ramps. The entire spectrum of the reference stimulus was shifted in 35−50 Hz increments to span six harmonics above and below the reference, limited to 200 Hz at the low-frequency end of the range (Fig. 2). All stimuli were presented in a random order, with 30 repetitions of each stimulus. The stimuli were presented at 43, 63, 73, or 83 dB SPL to either the contralateral ear or diotically. The 73 dB SPL stimuli were presented to a different set of neurons than the 43, 63, and 83 dB SPL stimuli; thus analysis of responses versus sound level were based only on responses to the 43, 63, and 83 dB SPL stimuli.

Rate profiles in response to the synthetic-timbre stimuli were calculated by averaging the firing rate over the duration of the stimulus, excluding a 50 ms onset. Synthetic-timbre rates profiles were plotted as a function of spectral peak frequency.

Temporal analyses included calculating period histograms at the 200 Hz spacing of the components and calculating vector strength to the 200 Hz periodicity of the stimuli. We also calculated vector strength for phase locking to integer multiples of 200 Hz. There were generally not static harmonics in these stimuli; however, we tested phase locking to the envelope periodicity. Analyses were performed to test for the presence of mode locking (Laudanski et al., 2010); briefly, we used surrogate spike trains compared with actual temporal responses to test if spike timing depended on both interspike interval structure and stimulus phase.

**Figure 3.**
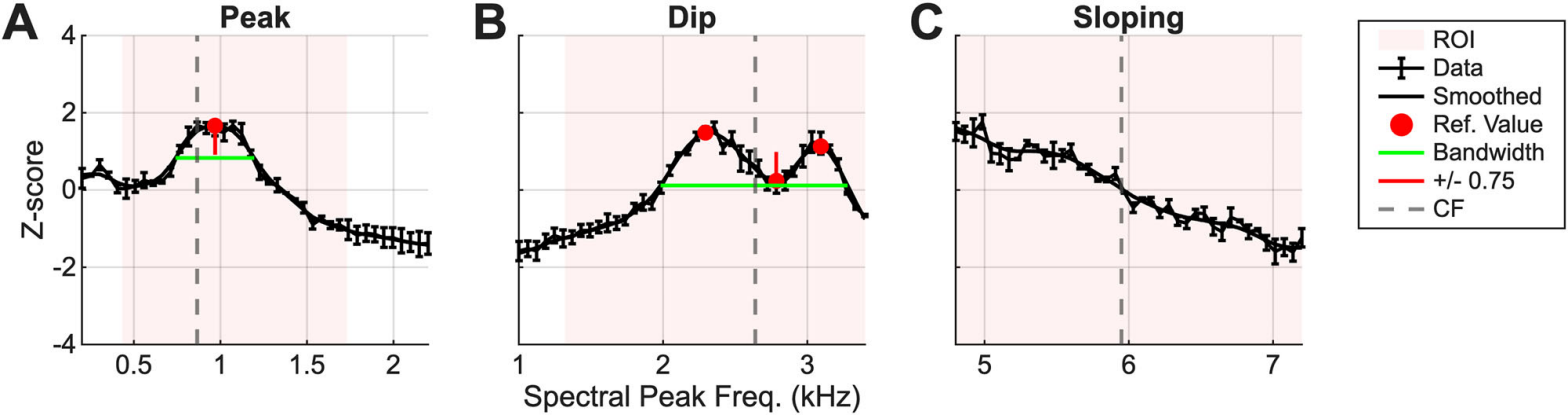
Examples of peak, dip, and sloping rate profiles, with quantification of peak and dip profiles. ***A***, Example rate profile with a peak near CF. Pink-shaded area represents region of interest (±1 octave around CF). Red dots indicate peaks/dips. The peak or dip (red circles) that was closest to CF (dotted gray line) was chosen as the reference value for determining bandwidth. The red vertical line indicates ±0.75 from the reference value, depending on peak or dip. The green line indicates bandwidth measurement. ***B***, Example rate profile with a dip. ***C***, Example of a sloping rate profile with no peaks or dips.

Synthetic-timbre rate profiles were separated into three categories that described the rate profile shape: peak, dip, and sloping (Fig. 3). First, average-rate profiles were smoothed and converted to *z*-scores using the mean and SD from the smoothed rate profile. Next, we identified peak profiles as those with a peak in the rate profile within ±1 octave around CF using the MATLAB function “findpeaks” with a minimum peak prominence set at 0.25. Dip profiles were identified by inverting the rate profile and applying the same criteria as for the peak analysis. Many neurons had both peaks and dips in the rate profiles; those neurons were categorized by which feature (peak or dip) was nearest to CF (Fig. 3*B*). Sloping neurons were categorized as having no peaks or dips within ±1 octave around CF. A salience metric, *Q*, was calculated for rate profiles that were categorized as peak or dip as follows:

$$Q=\frac{\;f_{p}}{BW_{0.75}},\;$$

where *f_p_* refers to the frequency at the peak or dip and BW_0.75_ refers to the bandwidth of the response at 0.75 *z*-score below the peak rate (or 0.75 *z*-score above the dip).

Neurons were also classified as sharpening, broadening, or unchanging over the sound level, depending on changes in *Q* as the sound level increased. A slope was determined using linear regression for *Q* values at 43, 63, and 83 dB SPL. If the slope was >0.03/dB, the neuron was classified as sharpening, if the slope was less than −0.03/dB the neuron was classified as broadening. A slope within −0.03 and 0.03/dB was determined to be unchanging.

An analysis of the synthetic-timbre rate profile was performed to test how the rate representation changed over the duration of the stimulus. The average-rate profiles were computed over two time windows, 50−150 ms and 200−300 ms. The salience metric, *Q*, was used to quantify changes in peaks or dips in the rate profiles over the duration of the stimulus.

A neural timbre-discrimination threshold was calculated for each IC neuron. First, rates and standard deviations of rates were linearly interpolated to include 600 steps between the lowest and highest spectral peak frequency. Then, the steepest slope between two consecutive spectral peak rates (*n*, *n* + 1) was found, and the adjusted *d*′ was calculated for those two rates:

$$d^{\prime }\;=\;\frac{\left|\;\mu_{2}-\mu_{1}\right|}{\sqrt{(\sigma_{1}^{2}+\sigma_{2}^{2})/2}}.$$

If the *d*′ was greater or equal to 1, then threshold was calculated using those two rates. However, if *d*′ was <1, the steepest slope between (*n*, *n* + 2) was then found. The process continued, sampling (*n*, *n* + i) where i = 1, 2, 3, …, 100, until either a *d*′ of one was found or no threshold could be calculated. Lastly, the frequency-discrimination threshold, in percentage of frequency, was calculated as follows:

$$\Theta =\;\frac{\Delta f}{\;f_{\mathrm{mid}}}\times 100,$$

where Δf is the difference in spectral peak frequency between the two rates and *f*_mid_ is the frequency at the midpoint of the two spectral peak frequencies. Neural thresholds were classified as improving, worsening, or unchanging over level using the slope from linear regression for thresholds at 43, 63, and 83 dB SPL. Similar to the previous classification, if the slope was >0.03/dB, the neuron was classified as a worsening threshold, a slope within +/−0.03/dB was classified as an unchanging threshold, and a slope less than −0.03/dB was classified as improving threshold.

Temporal thresholds were calculated using two methods. First, temporal thresholds were found based on the vector strength to the stimulus components, which were spaced 200 Hz apart, for the same 600 frequency steps that were used in the rate analysis. Second, a more agnostic approach computed the timbre-discrimination threshold using a rate-independent spike (RIS) distance metric (Satuvuori et al., 2017) using a previously published MATLAB package, SPIKY (Kreuz et al., 2015). Briefly, the RIS distance was calculated by measuring the similarity between two spike trains based on the relative timing of the spikes. A distance matrix was generated to represent the mean distances between all pairs of spectral peak frequencies. The adjusted *d*′ was calculated by comparing the distance across frequencies to the baseline distance between trials of the same frequency as follows: the reference distance (*µ*_1_) was defined as the mean distance between different trials of the same spectral peak frequency, and the comparison distance (*µ*_2_) was the mean distance between trials of two spectral peak frequencies. The distances were linearly interpolated to include 600 steps between the lowest and highest spectral peak frequency, and the same sliding-scale threshold search used for the rate thresholds was performed. This approach ensured that a threshold was only reached when the interstimulus dissimilarity significantly exceeded the variability present in repeated presentations of the same stimulus.

### Receptive field models

Linear, spectral receptive field models were fit to the data to predict neural rate profiles based on the synthetic-timbre stimulus spectra. Two models were tested, a single excitatory Gaussian and a difference of Gaussians (DoG) that included both excitatory and inhibitory components. The procedure and equations used to fit both models are described in Su and Delgutte (2020). Briefly, model predictions were calculated as the sum of stimulus spectral components, each weighted by the corresponding model magnitude, with the spontaneous firing rate of the neuron added as a constant. For the model with inhibitory weights, the prediction was half-wave rectified to avoid negative rate predictions. Model parameters were determined using the MATLAB function fmincon to minimize the mean squared error between the rate profile and the prediction. The result was a Gaussian or DoG model that provided the best model prediction fit to the neural rate profiles. An adjusted goodness-of-fit *R*^2^ metric (Su and Delgutte, 2020) was used to evaluate model performance while accounting for differences in the number of parameters in each model. Single-sided *F* tests were used to evaluate if the DoG model fit better than the Gaussian model, with the null hypothesis that both models fit the data equally well.

### Computational models for IC responses

Three IC models energy, same-frequency inhibition–excitation (SFIE), and BMSI were evaluated against synthetic-timbre rate profiles. Model predictions were made for each neuron in the dataset. Detailed implementations of these models are described in Fritzinger and Carney (2025). Briefly, the energy model consisted of a fourth-order gammatone filter with a bandwidth based on cat AN *Q*_10_ values (Carney and Yin, 1988) centered on the CF of the neuron. The SFIE and BMSI models both included an AN model with cat tuning parameters (Zilany et al., 2014). The SFIE model is a phenomenological model of BE and BS MTF IC neurons that accurately predicts AM sensitivity in IC neurons (Nelson and Carney, 2004; Carney and McDonough, 2019). This model includes SFIE, where the time course and amplitude of inhibition compared with excitation creates AM sensitivity. The BMSI model expands on the SFIE model by adding two parallel off-CF BS channels that inhibit the on-CF BS model neuron. The three inhibitory channels (one below CF, one at CF, and one above CF) constitute broadband inhibition. Depending on the inhibition strength, best modulation frequencies (BMFs), and off-CF frequencies chosen, BE, BS, hybrid, and flat MTFs can be simulated using this model configuration. The parameters used in the BE and BS broad inhibition model were from Fritzinger and Carney (2025) and were fit to responses to tones in wideband noise and to MTFs from 60 to 70 dB SPL. Model fits to data were evaluated using the variance-explained goodness-of-fit metric, *R*^2^. The *Q* value and thresholds calculated from neural rate profiles were also applied to the model rate profiles to compare trends in the model responses to those in the data.

### Statistics

A linear mixed-effects model was used to detect statistically significant changes in salience, log(*Q*), due to stimulus parameters and neuron characteristics. *Q* was log transformed for normality. Model selection was done using MATLAB functions LME and compare; then JASP (JASP Team, 2024, Version 265 0.18.3, computer software) was used for the remaining analyses. The fixed effects included MTF type, level (dB SPL), CF group, and presentation ear (contralateral or diotic). Neuron identity was added as a random effect, implemented as a random intercept, to account for heterogeneity in the neuron population. The model chosen included two-, three-, and four-way interactions between the four fixed effects. Model fit was assessed using visual examination of residuals and quantile–quantile plots in MATLAB. In JASP, models were analyzed using Type 3 ANOVAs using the Satterthwaite approximation. Other statistical methods used throughout this study include *t* tests, the Kruskal–Wallis test for nonparametric data, and comparisons of correlation coefficients and variance explained.

### Code accessibility

Custom MATLAB code for clustering data (Schwarz et al., 2012) available at https://www.urmc.rochester.edu/labs/carney/publications-code/spike-sorting-code.aspx. Custom MATLAB code for data analysis and modeling along with data files are available at https://osf.io/uyn56. Code is also available on GitHub at https://github.com/jfritzinger/FritzingerCarney2025-SynthTimbre.

## Results

We recorded responses of 188 neurons in the central nucleus of the IC in four awake Dutch-belted rabbits to synthetic-timbre stimuli. Responses to diotic synthetic-timbre presentations at 43, 63, 73, and 83 dB SPL were recorded in 153, 163, 82, and 152 of the 188 neurons, respectively. Responses to contralateral synthetic-timbre presentations at 43, 63, 73, and 83 dB SPL were recorded in 66, 70, 13, and 66 of the 188 neurons, respectively. The CFs of the dataset ranged from 320 to 9,236 Hz, with a median of 2,313 Hz (Fig. S1*A*). The MTF distribution was 45 BE (23.9%), 103 BS (54.8%), 24 hybrid (12.8%), and 16 flat (8.5%; Fig. S1*B*). BMFs of BE neurons ranged from 12 to 324 Hz with a median of 67 Hz, and worst modulation frequencies of BS neurons ranged from 2 to 435 Hz with a median of 78 Hz (Fig. S1*C*,*D*).

### Basic rate properties of responses to synthetic-timbre stimuli

**Figure 4.**
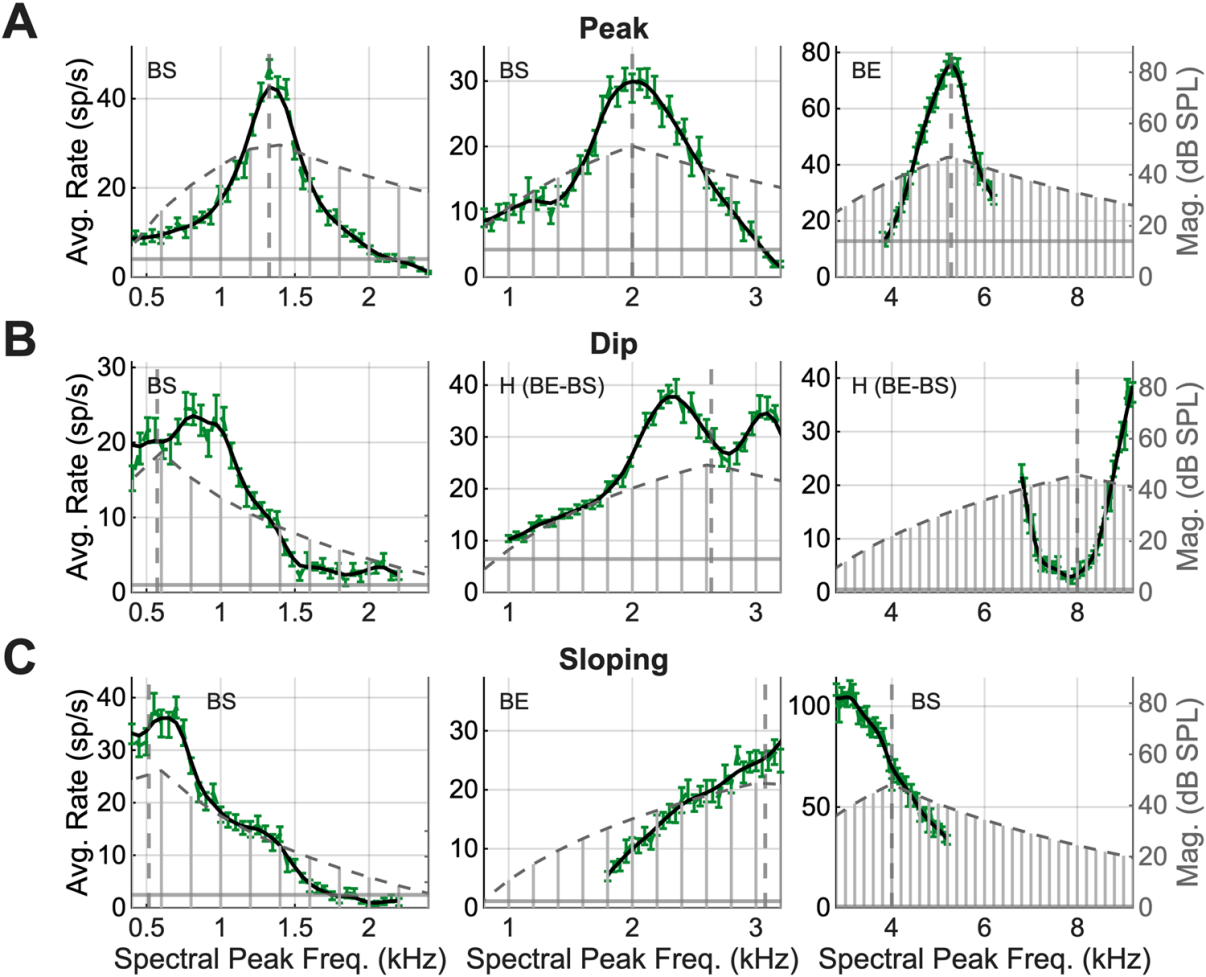
Average-rate responses for nine example neurons to synthetic-timbre stimuli at 63 dB SPL, with low, medium, and high CFs (left to right). ***A***, Three examples of responses with a peak near CF. Labels of MTF type in the top left corner of each plot. Data ± standard error of the mean (SEM; green), smoothed data (black), estimated CF (dashed gray line), and spontaneous rate (dark gray horizontal line). The stimulus spectrum is overlayed in gray with a gray-dotted line outlining the spectral envelope. Synthetic-timbre peak frequencies span 2,400 Hz for all examples. ***B***, Three responses with a dip near CF. ***C***, Three neurons with sloping responses.

Most IC neurons have rate MTFs that are enhanced or suppressed by a given band of modulation frequencies and vary with the modulation depth of AM stimuli (Langner and Schreiner, 1988; Krishna and Semple, 2000; Joris et al., 2004; Nelson and Carney, 2007; Kim et al., 2020). Depending on the MTF type, we hypothesized that the spectral peaks of synthetic-timbre stimuli are encoded by peaks or dips in the timbre rate profiles across populations of IC neurons. This hypothesis was tested by analyzing average-rate responses to stimuli that were spectrally shifted past CF to simulate population responses (Fig. 1). Each rate profile represents an inferred population response for neurons tuned at, above, or below the spectral peak frequency, and rate profiles were expected to be similar but reflected around CF compared with the population-model predictions (Fig. 1). Neural responses varied, and rate profiles were classified into three groups: peaks, dips, and sloping responses (Fig. 4). Peaked responses occurred in neurons at low (<2 kHz), mid (2–4 kHz), and high (4 + kHz) CFs (Fig. 4*A*, left, middle, right). Similarly, responses with dips or slopes occurred in neurons with a range of CFs (Fig. 4*B*,*C*). The response category was not always determined by the MTF type of the neuron. Examples of peak, dip, and sloping responses were present in all MTF types (Fig. 4), inconsistent with the strict hypothesis that the MTF type would completely determine the shape of the rate profile. Quantifications of the NF hypothesis for BE and BS responses are shown in the next section. Rate profiles were sharper than the stimulus envelope in all classifications.

### Temporal properties of responses to synthetic-timbre stimuli

**Figure 5.**
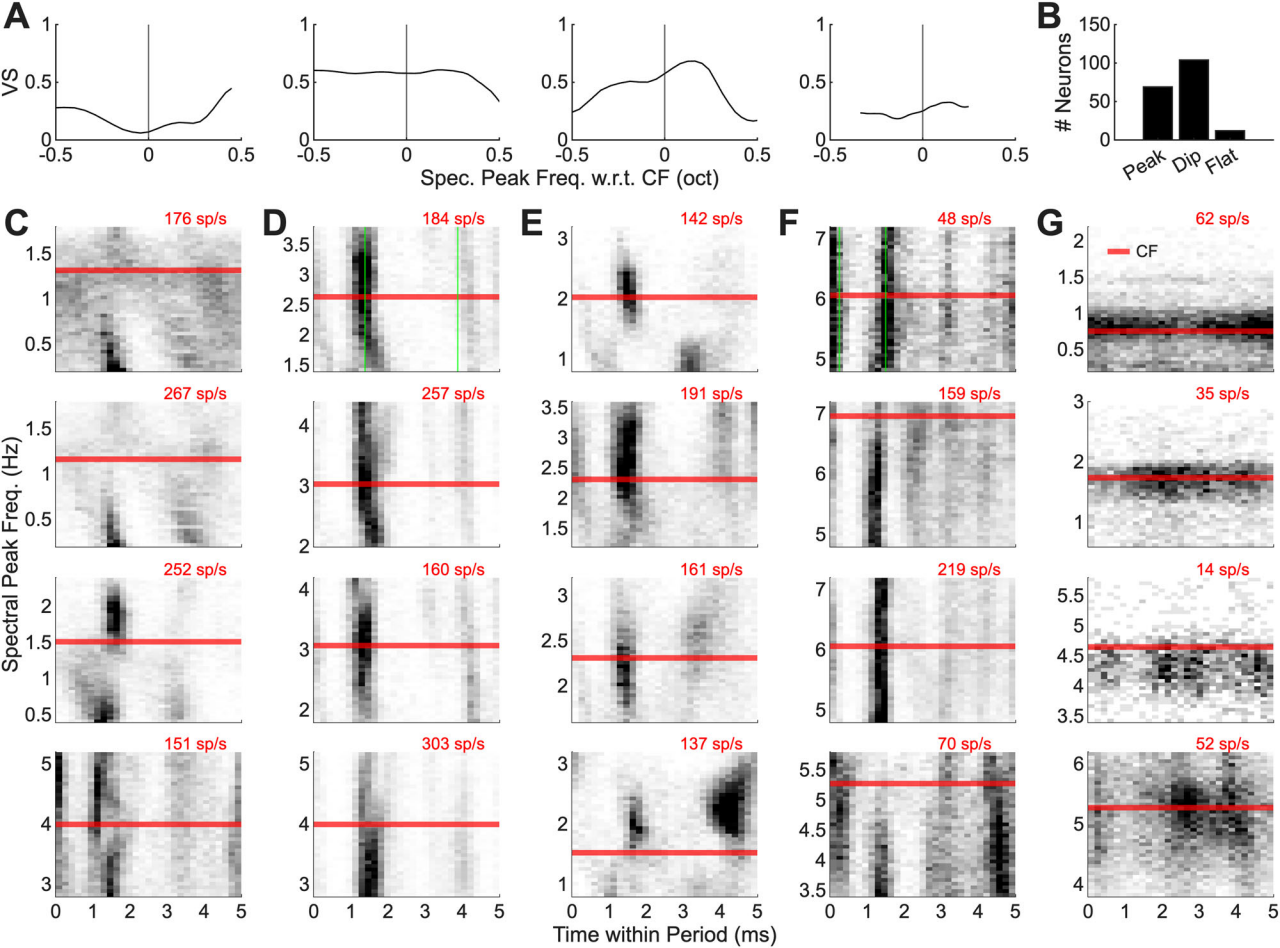
Temporal responses were influenced by phase locking to the 200 Hz spacing of the components and its harmonics. ***A***, Example vector strength calculations for the top example neuron in each column (***C–F***). Vector strength for G is near 0 and is not shown. ***B***, Histogram of vector strength profiles characterized as peak, dip, or flat. ***C–G***, Normalized period histograms for one period of 200 Hz for a range of peak frequencies for 20 example neurons to synthetic timbre, diotic, at 63 dB SPL. The red line indicates CF, and numbers in the upper-right corner of each plot are the maximum rate across all spectral peaks. Example neurons were grouped as follows: (***C***) decrease in phase locking near CF, (***D***) strong mode followed by 1–2 weaker modes, phase locking to 400 Hz (green), (***E***) mode(s) that decrease away from CF, (***F***) many modes, phase locking to 800 Hz (green), and (***G***) little or no phase locking.

Before continuing with a presentation of rate analyses, we will consider the temporal responses of IC neurons to the synthetic-timbre stimuli. Vector strength to the 200 Hz periodicity of the stimuli was calculated for responses to each spectral peak frequency to test the hypothesis that the vector strength decreases near the spectral peak (Fig. 1) due to reduced NF depth near the spectral peak. The vector strength profile would thus provide information about the spectral peak location. Vector strength varied in individual neurons (Fig. 5*A*), and many neurons had a dip in vector strength near the CF of the neuron (Fig. 5*B*). Period histograms for 200 Hz were plotted for all spectral peak frequencies to further scrutinize temporal response features (Fig. 5*C*–*G*).

The neuron response near CF was classified as peak, dip, or sloped based on vector strength. For the 63 dB SPL condition, responses of 55.2% of neurons supported the initial hypothesis that vector strength decreases when the spectral peak was near CF (*n* = 90/163; examples in Fig. 5*C*). An increase in phase locking to 200 Hz when the peak was near CF was observed in 37.4% of responses (*n* = 61/163; Fig. 5*E*; Fig. S2*C*). Finally, 7.4% of responses had vector strengths that were unchanged over the range of spectral peak frequencies tested (*n* = 12/163; Fig. 5*D*,*F*; Fig. S2*B*).

Neural thresholds of spectral peak discrimination were calculated based on changes in the vector strength to 200 Hz as the spectral peak was shifted in frequency, for the 63 dB SPL condition (Fig. S3*A*). Note that the stimuli varied from being harmonic to inharmonic as the spectrum was shifted with respect to CF; the harmonicity or inharmonicity changed the envelope depth of the stimulus. However, vector strength calculations did not vary between harmonic and inharmonic stimuli. Neural timbre-discrimination thresholds based on vector strength were poor, with only 8.6% of neurons (14/163 neurons) having a threshold <6%. In comparison, the human threshold for spectral peak discrimination is 4% (Allen and Oxenham, 2014). Additionally, 41.1% of neurons (67/163) had thresholds >50%. The large diversity in temporal responses and phase-locking patterns in these responses resulted in poor thresholds. Many neurons exhibited phase locking to integer multiples of 200 Hz (Fig. 5*C*–*G*; Fig. S2). The vector strength metric was reduced in neurons that phase locked to integer multiples of the 200 Hz periodicity of the stimuli. Based on this analysis, we concluded that the vector strength to 200 Hz was not monotonically related to spectral peak frequency for most neurons.

Despite these poor thresholds based on vector strength to 200 Hz, the temporal patterns were interesting and often exhibited phase locking. Significant phase locking to 200 Hz and integer multiples of 200 Hz at 63 dB SPL for at least 20 of the spectral peak frequencies presented was found in 53.4% of neurons (*n* = 98/163; Fig. 5*C*–*F*; examples, Fig. S2). Another 13.5% of neurons had significant phase locking to only integer multiples of 200 Hz (*n* = 22/163), and 6.7% of neurons had phase locking to 200 Hz but not to integer multiples (*n* = 11/163). The remaining 26.4% of neurons had little to no significant phase locking to 200 Hz or integer multiples of 200 Hz (*n* = 43/163; Fig. 5*G*; Fig. S2*E*). Out of the neurons that exhibited phase locking to integer multiples of 200 Hz, 54.1% of neurons had four peaks in the period histogram in each stimulus period for at least half of the spectral peak frequencies and phase locked to 800 Hz (*n* = 59/109; Fig. 5*F*; Fig. S2*D*). Metrics designed to test for mode locking of brainstem neurons (Laudanski et al., 2010) were calculated for the IC responses. These mode-locking metrics, which are based on interspike interval structure, were developed for and successfully applied to cochlear nucleus responses, which typically have higher rates than IC neurons. The rates of the IC neurons were too low to determine significant mode locking; no IC neurons had significant mode locking based on the Laudanski et al. (2010) metric.

A more agnostic approach to determine a spectral frequency threshold was determined using the RIS timing metric (Satuvuori et al., 2017). This approach measures temporal similarity between two spike trains without rate information and a *d*′ is computed based on spike train distance across spectral peak frequencies. Using this metric, only 13/163 neurons had a *d*′ >1, with none of the neurons having thresholds as sensitive as human thresholds (Fig. S3*C*,*D*). Together these results indicate that the analyses described above of the temporal responses alone did not provide reliable neural correlates for spectral peak discrimination in the IC, motivating the more in-depth analysis below of rate profiles in response to the synthetic-timbre stimuli.

### Rate profiles peaked when CF aligned with the nearest harmonic to the spectral peak

**Figure 6.**
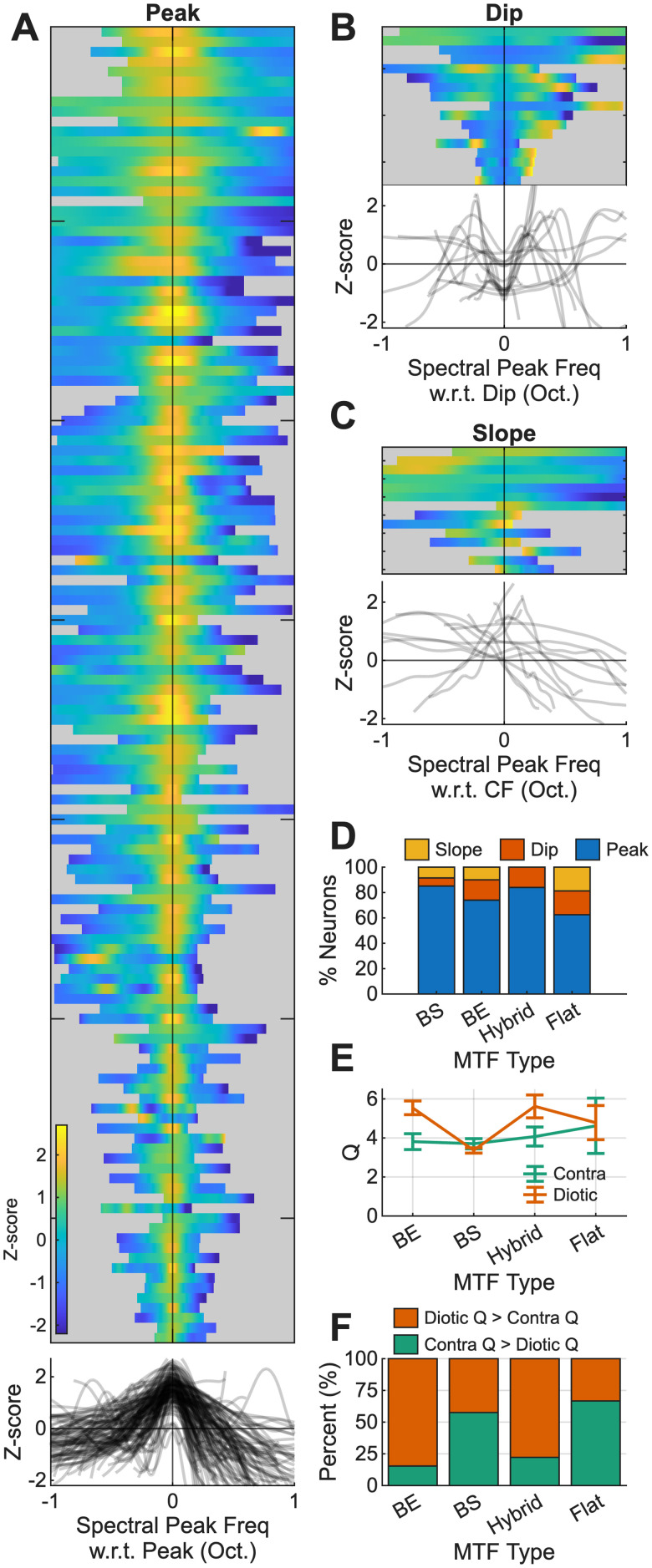
Population analysis to test the hypothesis that increases and decreases in rate (with respect to MTF type) capture the spectral peak. ***A***, Neuron responses to 63 dB SPL synthetic-timbre stimuli that are categorized with a peak near CF, ordered by CF with low CFs at the top and high CFs at the bottom. Gray traces (bottom) are all of the response profiles overlaid. ***B***, Neuron responses that feature a dip near CF. ***C***, Neuron responses that feature sloping profiles. ***D***, Proportions of peak, dip, and sloping responses for BE, BS, hybrid, and flat MTFs. ***E***, *Q* value plotted as a function of the MTF type, separated into binaural and diotic presentations. ***F***, The percentage of neurons that increase (orange) or decrease (green) in *Q* from diotic to contra stimulus presentation.

The population of average-rate profiles in response to the timbre stimuli that were shifted with respect to CF were analyzed to test the NF hypothesis in more depth. A salience metric, *Q*, was calculated to characterize the synthetic-timbre rate profiles. Neural rate profiles featured robust peaks, dips, and sloping responses. For the 63 dB SPL, diotic presentation, 81% (132/163) of rate profiles featured peaks near CF (Fig. 6*A*), 10% (17/163) featured dips near CF (Fig. 6*B*), and 9% (14/163) featured sloping responses (Fig. 6*C*).

The majority of responses featured peaks near CF, regardless of the MTF type (Fig. 6*D*). Thus, BS neurons were consistent with the hypothesis that responses would have peaks in the response due to NF profiles (Figs. 1, 6*D*). BE cells did not support the hypothesis that rates would decrease for spectral peaks near CF. Neurons with hybrid MTFs were generally expected to have a rate profiles similar to those of BE or BS types, depending on whether the MTF showed excitation or suppression at a modulation frequency that matched the 200 Hz periodicity of the stimulus. Additionally, neurons with flat MTFs were hypothesized to have flat rate profiles in response to synthetic-timbre stimuli. However, the majority of both hybrid and flat neurons also exhibited peaks in rate profiles. Overall, based on the initial hypothesis (Fig. 1), only neurons with BS MTFs were consistent with the simple NF hypothesis.

Next, the salience metric, *Q*, was calculated for every response with a peak or a dip (Fig. 3) to further characterize these neural responses and compare the sharpness of the synthetic-timbre rate profiles across neurons. There was no significant difference in salience (*Q*) between responses with peaks or dips (*p* = 0.5077, Welch’s *t* test). We hypothesized that salience would correlate with % MTF change (i.e., the MTF rate for unmodulated noise minus the maximum or minimum rate, for BE or BS, respectively), as previous studies had found using narrowband tone-in-noise stimuli (Fan et al., 2021). However, there was not a significant linear relationship between MTF strength and salience (BE, *p* = 0.7221; BS, *p* = 0.6853, linear regression). Interestingly, BE and hybrid neurons had significantly sharper salience in the diotic condition compared with the contralateral condition (Fig. 6*E*; *F*_(4,565)_ = 3.33; *p* = 0.010). A larger proportion of neurons with BE and hybrid MTFs had sharper responses to diotic compared with contralateral presentations, whereas BS and flat MTF responses were more evenly divided (Fig. 6*F*). These results suggest that the ipsilateral input to the IC, which is often inhibitory, sharpened responses to the diotic presentation of the stimulus but only for neurons with BE and hybrid MTFs. More experiments are required to clarify how the ipsilateral pathway impacts responses in these neurons. Overall, the results of these analyses suggested that the NF hypothesis is consistent with synthetic-timbre rate profiles for BS neurons, but not for neurons with other MTF types.

### Rate profiles were robust across a range of suprathreshold levels

**Figure 7.**
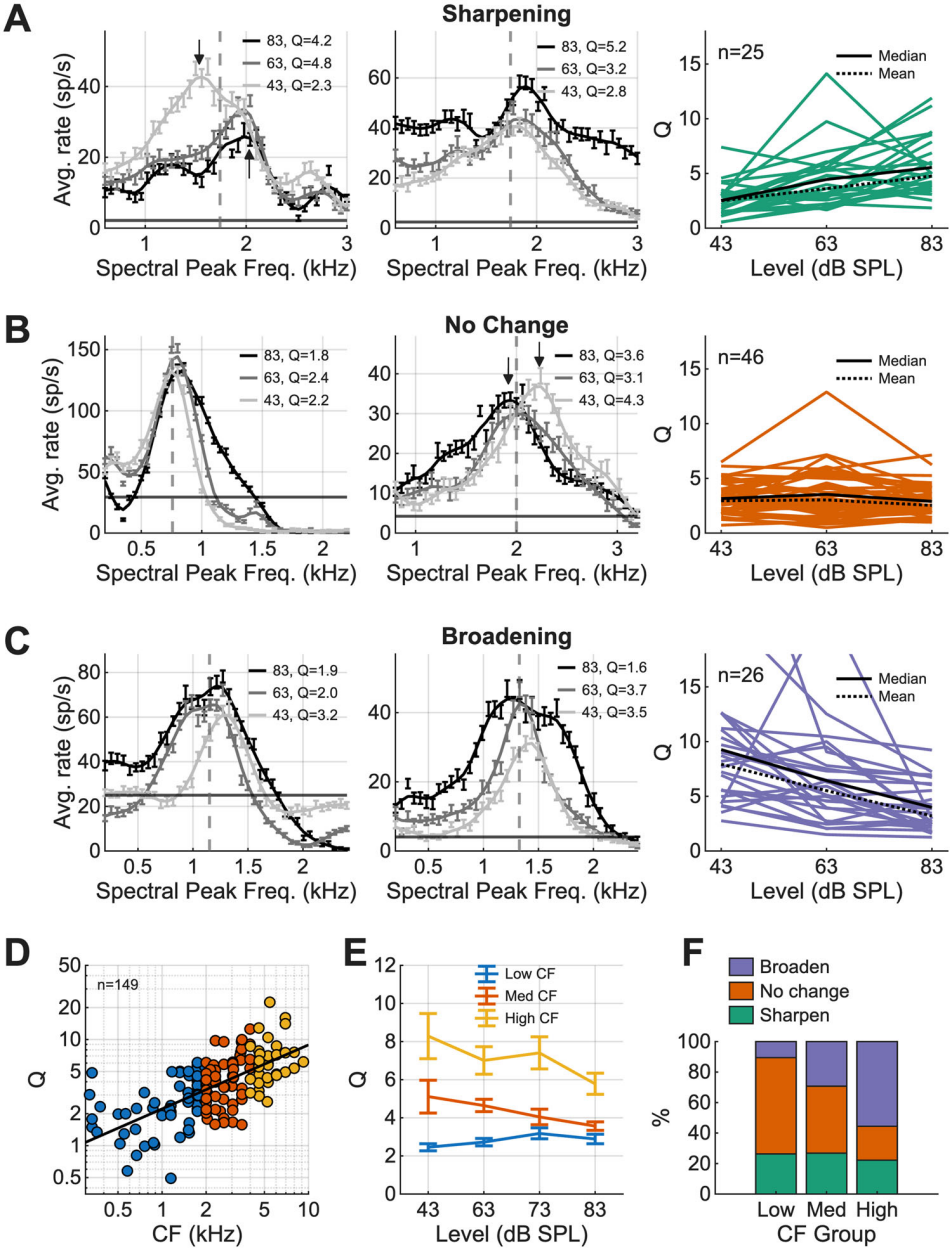
Analysis of neural responses over level. ***A***, Left, middle, Two example neuron responses with peaks near CF and arrows pointing to peak locations that have changed as level changes, (right) *Q* versus level for neurons that sharpen in *Q* as the level increases. ***B***, Same as ***A***, for neurons that do not change over level. Examples shown have peaks near CF with arrow. ***C***, Same as ***A***, for neurons that broaden in *Q* as the level increases, examples have peaks near CF. ***D***, *Q* values as a function of CF for the 63 dB SPL condition, split into three CF groups: low (blue), med (orange), and high (yellow). Trendline in black. ***E***, Significant interaction between the level and CF grouping in LMM; *Q* value plotted as a function of the level for three different CF groups. ***F***, Histogram showing the percentage of neurons that broaden, have no change, or sharpen in the *Q* value split into CF groups. The low CF group has a higher number of neurons that are unchanging, whereas the high CF group shows more units that decrease (broaden) in *Q*.

Although the NF hypothesis was only partially supported, the neural responses robustly encoded the spectral peak of the stimulus in the 63 dB SPL condition. We next tested the robustness of rate profiles in response to synthetic-timbre stimuli over suprathreshold levels to verify that the coding of the spectral peaks generalizes across levels. Although perceptual results for timbre discrimination across sound levels are not available, a robust model for timbre coding should function across a range of sound levels. Salience was calculated for all neurons with responses to diotic stimuli at 43, 63, and 83 dB SPL (*n* = 97). When pooling across all neurons, there was no significant change in *Q* as level increased (*p* = 0.4208, Kruskal–Wallis). However, individual neurons had heterogeneous responses, and many increased or decreased in *Q*. To quantify these changes, neurons were divided into three categories: sharpening, no change, and broadening (Fig. 7). Sharpening as the level increased occurred in 26% of neurons, often with an overall rate decrease as levels increased (*p* = 1.2245 × 10^−5^, Kruskal–Wallis; Fig. 7*A*). Neurons without large changes in *Q* made up 47% of neurons, and these rate profiles were mostly static as level increased (*p* = 0.4196, Kruskal–Wallis; Fig. 7*B*). Lastly, 27% of neurons broadened as the level increased (*p* = 8.3599 × 10^−5^, Kruskal–Wallis; Fig. 7*C*). Overall, despite heterogeneous responses across neurons, the population maintained robust spectral peak coding over the range of levels tested, with almost half of the neurons having little change in *Q* as the level increased. Salience increased (sharpened) as a function of CF for all three sound levels tested (Fig. 7*D*). There was a significant interaction between CF grouping and *Q* (Fig. 7*E*, LMM; *F*_(1,555)_ = 3.192; *p* = 0.026). However, *Q* did not change significantly over level for low, medium, or high CF groups (Fig. 7*E*, Kruskal–Wallis, low CF, *p* = 0.1542; medium CF, *p* = 0.1208; high CF, *p* = 0.1808). Additionally, when the proportions of broadening, sharpening, and unchanging neurons were divided into three categories based on CF, low CFs had a greater proportion of neurons that did not change in salience as the level increased (Fig. 7*F*). Other interesting changes over level include shifts in frequency of the largest peak of the neural response for some neurons (Fig. 7*A*,*B*, arrows). Original predictions based on the NF hypothesis (Fig. 1) did not hypothesize changes in neural excitation based on the sound level and instead predicted broadening as the level increased, as in Figure 7*C*. The mechanisms and perceptual implications of these shifts in frequency of peak excitation and changes in salience based on CF are unknown.

### Neural thresholds based on rate changes can explain human timbre-discrimination thresholds

**Figure 8.**
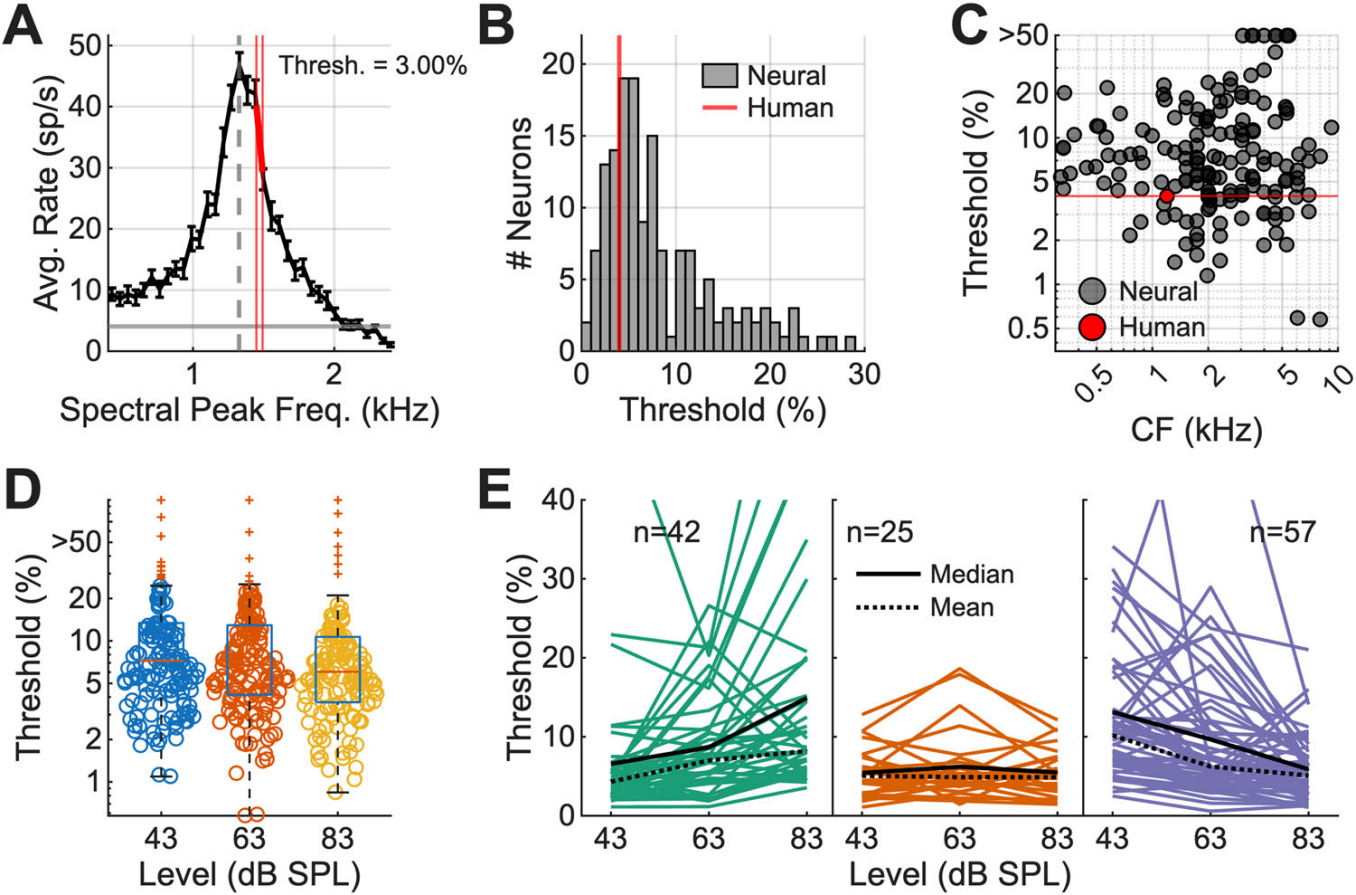
Timbre-discrimination thresholds for each neuron, for all sound levels and for diotic presentation. ***A***, Example neuron response: the thick red line indicates the steepest slope that yields a *d*′ ≥ 1. Thin red vertical lines indicate the spectral peak frequencies used for threshold calculation. $\mathrm{Threshold}=\;\frac{\Delta f}{\;f_{\mathrm{mid}}}\times 100,$ where Δ*f* is the smallest difference in spectral peak frequency for which *d*′ = 1 based on a difference in rate. ***B***, Histogram of thresholds, where the red line indicates human threshold at 1,200 Hz for musicians (Allen and Oxenham, 2014). ***C***, Thresholds as a function of CF. Human threshold (red circle) shown for reference. ***D***, Plot of all thresholds for diotic presentation as a function of the level. No significant differences between groups. ***E***, For the subset of neurons for which responses to all three levels (43, 63, and 83 dB SPL) were available, split into three categories: increasing (worsening) threshold, steady thresholds, and decreasing (improving) thresholds.

Next, neural discrimination thresholds for changes in the peak frequency of synthetic-timbre stimuli were compared with human timbre-discrimination thresholds from Allen and Oxenham (2014) to assess whether neural sensitivity in the IC could account for behavioral timbre discrimination. Timbre-discrimination thresholds in humans for a 200 Hz *F*_0_ and a 1,200 Hz peak were 4% of the peak frequency for musicians and 5% for nonmusicians (Allen and Oxenham, 2014). An example neuron with CF = 1,326 Hz had a discrimination threshold of 3.00%, lower than the behavioral thresholds (Fig. 8*A*). Across all neural threshold estimates in response to 63 dB SPL, diotic stimuli, 22.1% of neurons had thresholds lower than 4% (Fig. 8*B*). Neurons with a wide range of CFs (758–8,000 Hz) had thresholds lower than 4% (Fig. 8*C*). In comparison to neural thresholds calculated using vector strength to 200 Hz, 89.6% of neurons (146/163 neurons) had better thresholds using rate profiles. These neural thresholds indicate that spectral peak information encoded by the IC is sufficiently sensitive to support timbre discrimination, and additional pooling of information in a population response would likely provide additional robustness.

Given that the rate profiles were generally robust over level (Fig. 7) but often heterogeneous, we also examined whether neural discrimination thresholds were level invariant. Overall, there was no significant difference in thresholds at 43, 63, or 83 dB SPL (*p* = 0.0798, Kruskal–Wallis; Fig. 8*D*). Neurons were divided into three categories: neurons with improved thresholds made up 46% of the population (*n* = 57/124; *p* = 1.5792 × 10^−5^, Kruskal–Wallis), whereas neurons with worsening thresholds made up 34% (*n* = 42/124; *p* = 2.8456 × 10^−7^, Kruskal–Wallis), and neurons with unchanging thresholds made up 20% (*n* = 25/140; Fig. 8*E*; *p* = 0.9819, Kruskal–Wallis). Many IC neurons had improved thresholds based on the rate profiles as levels increased. The behavioral timbre-discrimination task has not been tested over level, and psychophysical thresholds cannot be directly compared. However, these neural threshold results are consistent with broader psychophysical findings that behavioral thresholds in speech intelligibility tasks improve as the level increases over a range of suprathreshold sound levels (Pickett, 1956; Studebaker et al., 1999).

### Spectral receptive field models with inhibition predicted synthetic-timbre rate profiles

**Figure 9.**
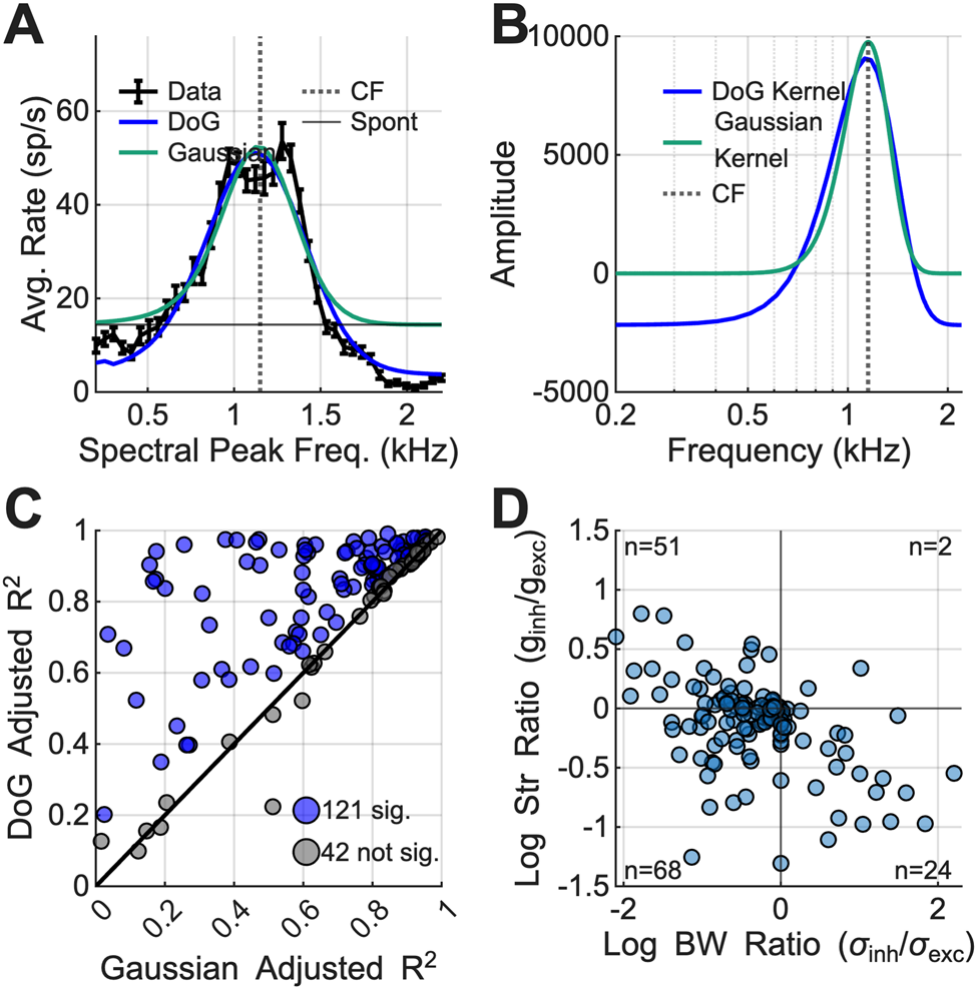
Gaussian versus DoG spectral receptive field model results to investigate the importance of adding broad inhibition. ***A***, Example DoG and Gaussian fit to an example neuron. ***B***, DoG and Gaussian receptive field model kernel for example shown in ***A***; kernel is multiplied and summed with the stimulus spectrum to obtain neural predictions seen in ***A***. ***C***, Comparison of adjusted *R*^2^ for Gaussian and DoG; significant increases in DoG *R*^2^ in blue. ***D***, Scatterplot of strength ratio versus bandwidth ratio for fitted DoG parameters.

Overall, IC rate profiles to the synthetic-timbre stimulus were robust and had timbre-discrimination thresholds that were similar to human thresholds, but the mechanisms behind these responses are unknown. Only BS responses were consistent with the NF hypothesis. To start examining the role of broad inhibition in these responses, spectral receptive field models, based on Gaussian or DoG functions, were fit to neural rate profiles. These models further characterized the synthetic-timbre rate profile shapes with minimal parameters describing excitatory and/or inhibitory components. Overall, the DoG model’s addition of an inhibitory Gaussian led to better predictions of rate profiles as compared with the pure excitatory Gaussian model (Fig. 9). An example neuron and the DoG and Gaussian fits to the synthetic-timbre rate profile shows how the added inhibitory term in the DoG model predicted rate below the spontaneous rate of the neuron, increasing model accuracy (Fig. 9*A,B*). Adjusted *R*^2^ values were calculated based on *R*^2^ and the number of parameters in the model (Fig. 9*C*). Seventy-four percent of model fits were significantly improved by adding the inhibitory term (single-sided *F* tests; Fig. 9*C*). The shapes of the DoG model fits were analyzed by comparing the ratio of inhibitory and excitatory strength to the inhibitory and excitatory bandwidth parameters (Fig. 9*D*; Fritzinger and Carney, 2025). Fits in the lower right quadrant (*n* = 24) represent models with broad, but weak, inhibition (Fig. 9*D*). Fits in the left upper and lower quadrants (*n* = 51; *n* = 68, respectively) represent models with narrow inhibition that either have a single inhibitory sideband or have double peaks with a dip in the middle of the response (examples, Fig. 4*B*). Eighteen percent of DoG models featured broad inhibition (*n* = 26/145). Narrow inhibition also improved fits for neurons with multiple peaks and single suppressed sidebands (*n* = 119/145), emphasizing the heterogeneity in rate profiles. These results suggest that inhibition played a role in responses to synthetic timbre, which was most clear in responses that decreased below spontaneous rate. However, for responses with double peaks, it was possible that other factors besides inhibition impacted those responses.

### IC models predicted synthetic-rate profiles at one level but failed to describe trends over level

Lastly, computational models featuring NF sensitivity and AM-tuned broad inhibition were tested to determine which mechanism better accounted for the neural data. Three IC models were used to predict the rate profiles in response to synthetic timbre: energy, SFIE, and AM-tuned broad inhibition models. The SFIE and BMSI models reliably predicted BE and BS MTFs [SFIE, Nelson and Carney (2004); Carney and McDonough (2019); BMSI, Fritzinger and Carney (2025). The SFIE model tested the simple NF hypothesis (Fig. 1), whereas the BMSI model added NF-sensitive off-CF channels. The energy model was included as a baseline; however, this model cannot replicate AM sensitivity and thus MTFs. Based on the DoG results and the finding that only BS neurons were consistent with the NF hypothesis, we predicted that the BMSI would predict neural responses most accurately.

**Figure 10.**
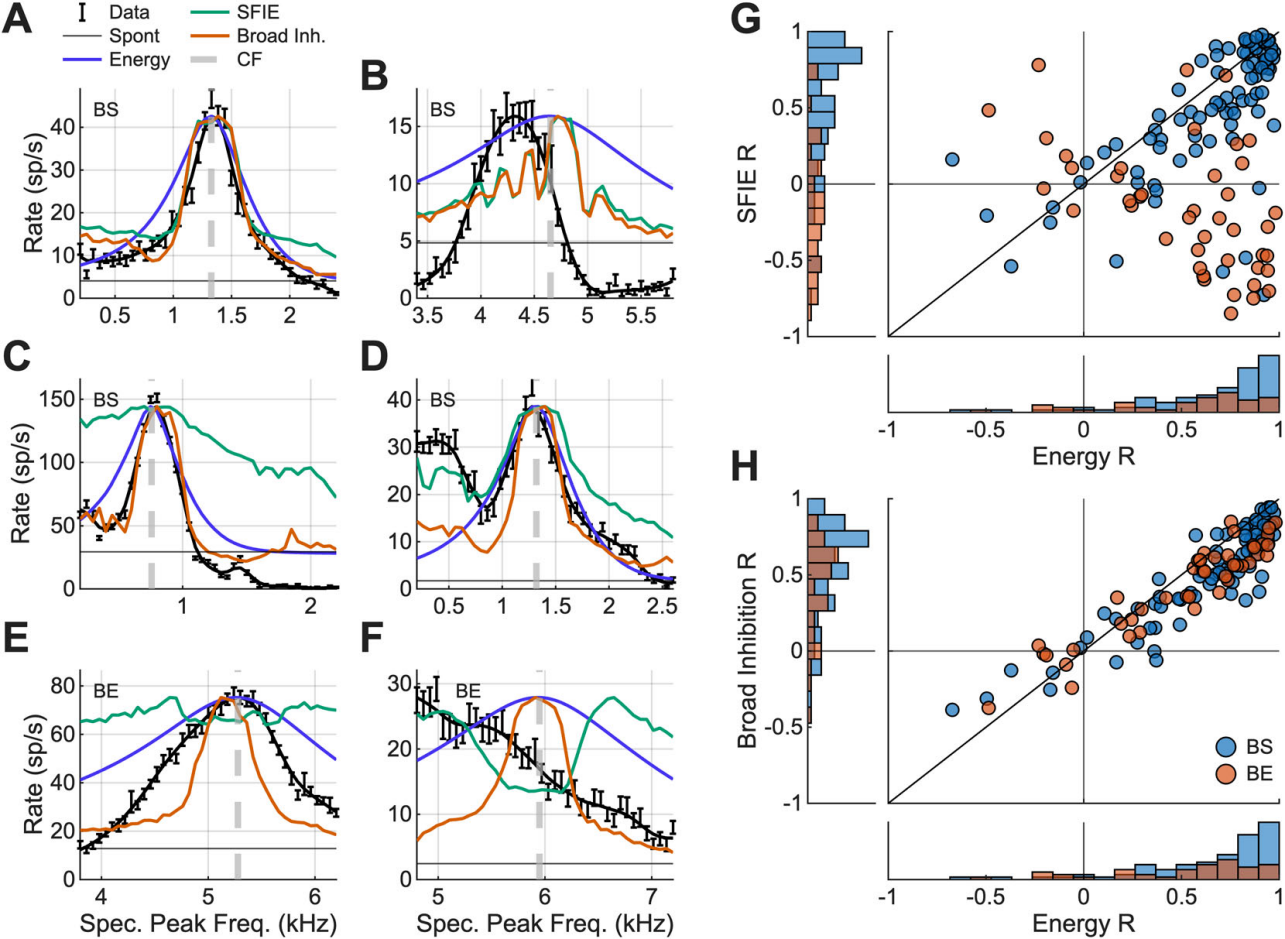
Rate profiles for six example neurons at 63 dB SPL and model predictions for energy, SFIE, and broad inhibition models. ***A***, BS neuron with responses that are predicted well by models. ***B***, BS neuron with poor model predictions. ***C***, BS neuron with inhibitory area consistent with broad inhibition model. ***D***, BS neuron with “dip” in rate off-CF, predicted by SFIE and broad inhibition models. ***E***, BE neuron with acceptable predictions from energy and broad inhibition. ***F***, BE neuron with poor model predictions. ***G***, Energy and SFIE model correlations to neural data for BE (red) and BS (blue) neurons, with histograms showing distribution for BE and BS neurons. ***H***, Same as ***G*** but for broad inhibition and energy model comparison.

These models were run for each neuron with a BE or BS MTF for the 63 dB SPL, diotic condition. Noise MTFs were also used as an input to the SFIE and BMSI models to check that the models predicted both synthetic-timbre rate profiles and also BE and BS MTFs. The SFIE model correctly predicted MTF shape for 93% of neurons, and the BMSI model correctly predicted MTF shape for 49.2% of neurons, with the error of predicting a flat MTF instead of BS. Neural responses to the synthetic-timbre stimulus were split into six categories based on model responses, with examples from each category in Figure 9*A*–*F*. The first category was neural responses that could be predicted by all three models. Twenty-eight percent of neurons were in this category; all of these neurons had BS MTFs, and all three models had an *R*^2^ (calculated between each model and neuron response) above 0.4 (*n* = 36/127; Fig. 10*A*). Second, for 35% of neurons, often with BS MTFs and higher CFs, all models were poor predictors of the neural responses and had *R*^2^ values below 0.4 (*n* = 45/127; Fig. 9*B*). SFIE and BMSI model responses often had a salience, *Q*, that is too sharp at high CFs (Fig. 10*A*). The third category consisted of 10% of neurons where BMSI model predictions were better than other models, often because the broad inhibition allowed model rates to decrease below the spontaneous rate (*n* = 13/127; Fig. 10*C*). The fourth category consisted of neurons with complex responses with multiple peaks, where BMSI and SFIE models outperformed the energy predictions (7%, 9/127; Fig. 10*D*). Another response we often saw were BE neurons that were poorly predicted by the SFIE model, which predicts a “dip” in the rate near CF, but better predicted by energy and BMSI (8%, 10/127; Fig. 10*E*). The last category was sloping responses (11%) where all models failed to predict sloping responses (14/127; Fig. 10*F*). Variance explained for each example neuron model response is detailed in Table 1. There was a large heterogeneity across neural responses, which could not be fully predicted by any of the IC models tested.

**Table 1.** Variance explained by the three model types for the six neuron examples in Figure 8

Neuron	SFIE *R*^2^	Energy *R*^2^	Broad inhibition *R*^2^
A	0.89	0.90	0.89
B	0.19	0.35	0.22
C	0.71	0.92	0.89
D	0.71	0.47	0.59
E	0.04	0.96	0.72
F	0.02	0.06	0.04

The correlation, *R*, of a model prediction and the synthetic-timbre rate profiles for each neuron was used to compare model performance. Neurons were separated into BE and BS MTFs, and predictions were compared for SFIE and energy models for the 63 dB SPL synthetic-timbre condition (Fig. 10*G*). SFIE and energy predictions for BS neurons were relatively accurate; however, the SFIE model failed to predict BE responses (Fig. 10*G*). Next, the BMSI and energy models were compared (Fig. 10*H*). Both models performed well, with energy having a slightly higher *R* overall (mean *R*_energy_ =  0.6008; mean *R*_broad inhibition_ = 0.4847). The BMSI model better predicted BE neurons compared with the SFIE model, consistent with the hypothesis that NF-sensitive AM–tuned inhibition impacts the coding of these responses.

Period histograms from the energy, SFIE, and BMSI models were also compared with neural data (Fig. 5, Fig. S4). SFIE model period histograms exhibited phase locking away from the spectral peak and a lack of phase locking at the spectral peak (Fig. S4*A*). The BMSI model (Fig. S4*B*) also exhibited phase locking away from the spectral peak, whereas the energy model (Fig. S4*C*) failed to predict any phase locking. None of these model responses had the complexity in temporal responses that were seen in the physiological data.

**Figure 11.**
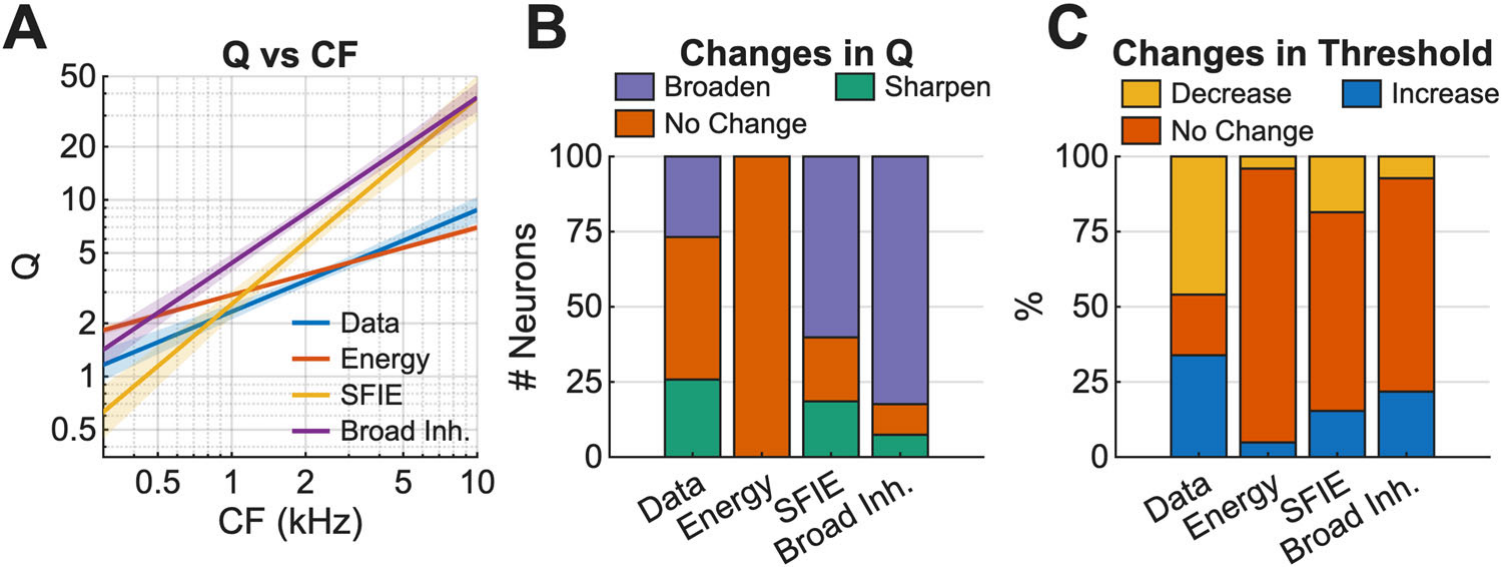
Evaluations of *Q* versus CF and trends in *Q* and threshold over sound level for energy, SFIE, and broad inhibition models. ***A***, Salience, *Q*, trendlines for data and models as a function of CF. ***B***, Broadening, sharpening, and unchanging Q calculated as level increases for data and three models. ***C***, Changes in neural discrimination thresholds for data and three models as the level increases.

Changes in Q as the sound level increased were calculated for model predictions of each neuron and were compared with trends in the neural data to further test these computational models. First, we compared the relationship of *Q* with CF in the neural data (Fig. 7*D*) and model predictions (Fig. 11*A*). The SFIE and BMSI model predictions had steeper slopes in the synthetic-timbre rate profiles than the neural data and the energy model predictions (Fig. 11*A*). Model responses for each neuron were classified into broadening, sharpening, or unchanging categories (Fig. 11*B*). The energy model predicted no change in *Q* versus level, whereas some SFIE and BMSI model neurons predicted broadened rate profile peaks as the level increased. No model matched the distribution of sharpening, broadening, and unchanging neurons in the data: the energy model overestimated the number of unchanging neurons, and the SFIE and BMSI models overestimated the number of broadening neurons.

Last, we calculated the changes in rate-based neural timbre-discrimination threshold as level increased for each of the models. For all three models, the majority of fits to the neurons had unchanging thresholds versus level, whereas more neurons had thresholds that decreased (improved) in threshold with level (Fig. 11*C*). These results emphasize that although rates can be predicted at one sound level with good accuracy, changes over level in the model responses did not match the trends in the data, and improvements to the models are needed.

Overall, the SFIE model and BMSI model accurately predicted responses in BS neurons, though the BMSI model sometimes failed to produce a BS MTF. For BE neurons, the BMSI model was more accurate than the SFIE model. The energy model, alternatively, was able to predict both BE and BS responses but failed for more complex responses and failed to predict AM sensitivity. All models struggled to predict changes in *Q* and threshold with level. These results suggest that the NFs may be critical for encoding of synthetic-timbre stimuli, but importantly both on- and off-CF NFs may impact IC responses.

## Discussion

Spectral peak encoding was investigated by recording single-neuron responses in the central nucleus of the IC in awake rabbits. Using a novel frequency-shifting paradigm, a population response was inferred from responses of a single neuron to a harmonic stimulus with a triangular spectral peak. Results support the hypothesis that spectral peaks in the rate profiles are sufficient to explain human thresholds in a timbre-discrimination task. Many neurons had robust rate profiles over several suprathreshold sound levels, and neural discrimination thresholds improved as the level increased.

One goal of this study was to investigate mechanisms of encoding these stimuli. The original NF hypothesis was only consistent with BS neuron responses. However, the BMSI model better predicted BE rate profiles. These findings are consistent with the idea that the encoding of wideband stimuli recruit off-CF channels that alter on-CF encoding of complex sounds (Fritzinger and Carney, 2025). Previous work comparing responses to tones in narrowband and wideband noise found AM-tuned broad inhibition was necessary to explain IC responses to tones in wideband noise, whereas responses to tones in narrowband noise were consistent with on-CF NF hypotheses (Fan et al., 2021; Fritzinger and Carney, 2025). Overall, these neural and modeling results for synthetic-timbre responses suggest an expanded NF mechanism, where NFs impact on- and off-CF pathways to contribute to neural coding.

Temporal responses to the synthetic stimuli were more complicated than initially predicted, leaving an open question about temporal coding of these stimuli in the IC. Interestingly, IC responses to a flat magnitude-spectrum harmonic complex with *F*_0_ = 200 Hz had a mean vector strength to *F*_0_ of 0.5 (Su and Delgutte, 2019). The mean vector strength to *F*_0_ for the synthetic-timbre stimuli was ∼0.25, suggesting a possible difference in temporal code between the flat and triangular-shaped harmonic complexes. The phase-locked responses also leave open questions about how and why lower harmonics are encoded in the IC.

Another impact on these IC rate profiles may be subcortical efferent-system feedback. Medial olivocochlear neurons receive input from cochlear nucleus and IC neurons and project to outer hair cells across frequency channels (Brown, 2014). The rate representation in the IC to these timbre stimuli may be sharpened by efferent feedback (Carney, 2024). We found that the sharpness of rate profiles in response to synthetic-timbre stimuli increased, decreased, or remained unchanged over the time course of the stimuli in about equal proportions, and this effect did not depend on the MTF type (Fig. S5). However, this result could be due to the 300 ms duration of the stimulus; a longer duration stimulus may have larger changes in rate due to efferent effects.

### Comparisons to vowel and timbre studies

Timbre results can be compared with IC neural responses to harmonic stimuli with triangular spectra meant to approximate spectral peaks or formants in vowels. These stimuli differ in the steepness of the spectral slope: often the vowel approximations use slopes of 200 dB/oct (Lyzenga and Horst, 1995; Henry et al., 2017), whereas the slope in our stimuli was 24 dB/oct. Neural timbre-discrimination thresholds based on rate profiles were generally consistent with neural vowel-discrimination thresholds in quiet (Henry et al., 2017). However, neural responses to synthetic vowels in the budgerigar supported on-CF NF mechanisms: BE neurons had decreased rate responses at the spectral peak. This result differs from the neural responses to synthetic timbre reported here. One possible explanation is the difference in bandwidth of these stimuli. For a simpler stimulus, tones in narrowband and wideband noise (Fan et al., 2021; Fritzinger and Carney, 2025), on-CF NF mechanisms are supported in narrowband studies, but off-CF mechanisms are necessary for wideband sounds. Additionally, Henry et al. (2017) found neural discrimination thresholds based on timing were needed to explain behavioral thresholds in noise, and while we did not test timbre stimuli in noise, the timing information in the IC for these stimuli were rich and varied (Fig. 5).

### Modeling

The DoG spectral receptive field model with broad inhibition predicted responses more accurately than an excitatory-only Gaussian model (Fig. 11). This finding is consistent with an implementation of a DoG spectral receptive field model for flat harmonic tone complexes (Su and Delgutte, 2020). However, DoG models were fits of a function directly to the synthetic-timbre rate profile and did not address the underlying mechanisms governing IC responses.

Neural models that included sound-driven AN responses as input were also explored in this study. The BMSI model was an improvement over the SFIE model and predicted rate profiles in neurons with both BE and BS MTFs (Fig. 9). Though the energy model had comparable fits to simple responses to synthetic timbre, the BMSI model also predicted MTFs and responses to tones in narrowband and wideband noise (Fritzinger and Carney, 2025).

The BMSI and SFIE models included an AN model with cat tuning parameters. These tuning parameters influence the sharpness of the rate response peak near the spectral peak. For example, using human tuning parameters resulted in a half-height bandwidth of the peak response in BS neurons equal to 290 Hz (Maxwell et al., 2020), whereas the half-height bandwidth using cat tuning was 656 Hz. Cat tuning parameters were used in this study instead of an implementation of rabbit tuning (Su and Delgutte, 2019) due to limited information about the rabbit periphery.

Limitations of the current IC models include inaccurate period histograms and trends in rate responses over the sound level. Many neurons phase locked to harmonics, in addition to the 200 Hz spacing of the components, but the models did not exhibit phase locking to harmonics except at very low fundamental frequencies (data not shown). Twenty-seven percent of neurons (*n* = 49/184) had decreased vector strengths and phase locking to harmonics when the peak harmonic was near CF, consistent with the models, but 73% of neurons (*n* = 135/184) had responses that the models could not predict. For the SFIE and BMSI models, most predictions had *Q*s that broadened as the level increased, in part due to AN saturation occurring more broadly across a range of CFs as the level increases. For all three models, most responses for models fit to neurons did not have level-dependent thresholds, whereas many neurons had thresholds that improved as the level increased.

A new IC model is needed to accurately predict response timing and changes with the level, as the limitations in the current models are not easily fixed. One option for a new IC model is a conductance-based model (Gerstner, 2001). Current SFIE and BMSI models use two alpha functions to model excitation and inhibition, whereas conductance-based models could provide more flexibility in model structure.

### Differences in synthetic and natural harmonic sounds with timbre

The synthetic-timbre stimulus is simplified compared with natural sounds with timbre, and encoding natural instrument spectra may be more complex. For example, the amount of energy at low harmonics differs: most instruments have strong fundamentals and low harmonics (for examples, see Siedenburg et al., 2021), whereas the synthetic-timbre stimulus used here had a weak or missing fundamental. This difference in energy at low frequencies may affect both temporal and rate coding, specifically for suprathreshold sounds. For example, AN fibers with higher CFs respond to low frequencies in the tail of the tuning curve at higher sound levels, which could alter the stimulus representation in these AN fibers and change the NF patterns (Carney, 2018). Low-frequency harmonics would alter IC firing rates similarly because IC response maps are often broad at high sound levels. The phase locking in the IC to the 200 Hz *F*_0_ could also be strengthened due to the higher magnitude *F*_0_ typical in natural stimuli.

Another key difference between synthetic and natural stimuli is the phase of the harmonics. The synthetic-timbre stimuli had harmonics added in sine phase. Vowels and instrument sounds have harmonic components with phase delays based on the resonances in the vocal tract or instrument body (Wolfe et al., 2009). These phase changes result in fast frequency sweeps, called chirps. Neurons in the IC are selective for upward or downward chirps and chirp velocity, and thus chirps in natural sounds would also alter IC responses (Mitchell et al., 2023).

### Implications of hearing loss

A motivation for studying spectral peak and timbre encoding is hearing loss: the perception of the sound spectrum is altered by hearing loss and in cochlear-implant users (Gfeller et al., 2002; Emiroglu and Kollmeier, 2008; Kong et al., 2011, 2012; Chintanpalli et al., 2016). Perceptual studies in which listeners identify just-noticeable differences between morphed instruments or identify vowels in concurrent-vowel experiments report deficits in instrument and vowel identification in listeners with hearing loss (Emiroglu and Kollmeier, 2008; Chintanpalli et al., 2016). Possible mechanisms related to perceptual deficits include loss of cochlear sensitivity due to outer-hair-cell damage and loss of inhibition due to aging (Caspary et al., 2008). Loss of cochlear sensitivity, for example, could lead to decreases in peak rates at the spectral peak of IC cells due to reduced contrast in NF profiles (Carney, 2018), whereas loss of inhibition could alter AM sensitivity and reduce broad inhibition mechanisms for AM sensitivity. Future work studying IC encoding of spectral peaks after hearing loss is critical to understand how IC representations are degraded in hearing loss.
